# Emotional well-being in children and adolescents treated with atomoxetine for attention-deficit/hyperactivity disorder: Findings from a patient, parent and physician perspective using items from the pediatric adverse event rating scale (PAERS)

**DOI:** 10.1186/1753-2000-2-11

**Published:** 2008-05-28

**Authors:** Peter M Wehmeier, Alexander Schacht, Martin Lehmann, Ralf W Dittmann, Susan G Silva, John S March

**Affiliations:** 1 Lilly Deutschland, Medical Department, Bad Homburg, Germany; 2 Department of Child and Adolescent Psychosomatic Medicine, University of Hamburg, Germany; 3 Duke University Child and Family Study Center, Duke University Medical Center, Durham, N.C., USA

## Abstract

**Background:**

The objective of this analysis was to measure changes in items on the Pediatric Adverse Event Rating Scale (PAERS) that relate to emotional well-being of children and adolescents with Attention-Deficit/Hyperactivity Disorder (ADHD) during treatment with atomoxetine for up to 24 weeks from the perspective of the patient, the parent, and the physician.

**Methods:**

Patients aged 6–17 years with ADHD were treated with atomoxetine (target dose 1.2 mg/kg/day). In the two studies on which this secondary analysis is based the PAERS was used to assess the tolerability of atomoxetine in children and adolescents. This scale has a total of 48 items. The ten items that reflect emotional well-being were selected to measure changes over time from a patient, parent, and physician perspective.

**Results:**

421 patients were treated with atomoxetine. 355 patients completed the 8-week treatment period, and 260 patients completed the 24-week treatment period. The ten items that reflect emotional well-being were grouped in five dimensions: depressed mood, self-harm, irritability/agitation, drowsiness, and euphoria. The scores of these dimensions decreased over time, both from a patient as well as from a parent and physician perspective. Only the dimension self-harm was extremely low at baseline and stayed low over time. The mean scores for the ten items depended on the rater perspective.

**Conclusion:**

The emotional well-being of children and adolescents with ADHD improved in terms of depressed mood, irritability/agitation, drowsiness, and euphoria during treatment with atomoxetine for up to 24 weeks.

## Background

Attention-deficit/hyperactivity disorder (ADHD) is a disorder characterized by inattention, impulsivity, and hyperactivity that affects 3–7% of school-age children [1]. ADHD is usually associated with significant impairment of cognitive and psychosocial functioning [2,3] and can have a significant impact on the emotional well-being [4-6] and the quality of life (QoL) of both patients and their families [7-12].

Psychostimulants and behavioral therapy are known to be effective in the treatment of ADHD, as reported in the MTA study [13]. Atomoxetine is a non-stimulant treatment option for ADHD [14,15], for which efficacy and tolerability in children and adolescents have been demonstrated in a number of randomized, placebo-controlled trials [16-19], supported by a recent meta-analysis [20]. In most of these studies, questionnaires such as the ADHD-Rating Scale (ADHD-RS) [21,22] or the Clinical Global Impression (CGI) [23,24] have been used as outcome measures for the core symptoms of ADHD. Other questionnaires such as the Child Health Questionnaire (CHQ) [25] or the Child Health and Illness Profile, Child Edition (CHIP-CE) [26] assess aspects of ADHD that go beyond the core symptoms of the disorder and reflect various dimensions of health-related quality of life. However, such questionnaires are often rated by the investigator alone, resulting in an assessment from one perspective only. Therefore, several studies have attempted to compare the perspectives of the various individuals involved, such as the patient, the parent, or the physician, as these perspectives have been shown to differ [12,27]. The newly devised Global Impression of Perceived Difficulties (GIPD) is one such instrument with which the three perspectives can be compared [28,29]. The Pediatric Adverse Event Rating Scale (PAERS) also allows the comparison between patient, parent, and physician perspectives, although it was designed to capture the tolerability of medication rather than efficacy [30].

This report is based on a secondary analysis of data from two almost identical multi-center, single-arm, open-label studies in two different age groups (children and adolescents). These studies were designed to investigate the quality of life in patients with ADHD treated with atomoxetine as reflected by the degree of difficulties perceived by patients, parents and physicians [28,29]. The two studies were undertaken to address the need for further research on evidence-based psychopharmacological treatments in children and adolescents [31]. One of the aims of the two studies on which this post-hoc analysis is based [28,29] was to assess the tolerability of atomoxetine in these patients and compare the tolerability as perceived from the three perspectives (patient, parent, physician) using the Pediatric Adverse Event Rating Scale (PAERS). The PAERS is a 48-item questionnaire designed to assess any type of adverse event occurring in pediatric patients who are treated with psychotropic medication, especially as participant in clinical trials, and was developed as part of the Child and Adolescent Psychiatry Trials Network (CAPTN) [30,32-34]. The response captures the severity of 48 adverse event items on a five-point Likert scale (0–4).

The main assumption of this post-hoc analysis was that 10 of the 48 items of the PAERS are directly related to the patients' emotional state and can therefore be considered to reflect the patient's emotional well-being. Based on this assumption, the hypothesis of this analysis was that the emotional well-being of children and adolescents with ADHD responds well to treatment with atomoxetine as reflected by the 10 items of the PAERS directly related to the patient's emotional state. Differences between the three perspectives (patient, parent, physician) were also explored.

## Methods

### Study design and procedures

This is a secondary analysis of data from two almost identical multi-center, single-arm, open-label studies in two different age groups (children and adolescents) that were designed to investigate the quality of life in patients with ADHD treated with atomoxetine as reflected by the degree of difficulties perceived by patients, parents and physicians [28,29]. Patients were recruited from child and adolescent psychiatric and pediatric practices and outpatient clinics throughout Germany. Patients aged 6–17 years with ADHD as defined in the Diagnostic and Statistical Manual of Mental Disorders, Fourth Edition, Text Revision (DSM-IV-TR) [1] were eligible for the studies. The diagnosis was confirmed using the "Diagnose-Checkliste Hyperkinetische Störungen" (Diagnostic Checklist for Hyperkinetic Disorders), a structured instrument which is routinely used for the diagnostic assessment of ADHD in Germany [35]. The items of this instrument correspond to those of the ADHD-RS [21,22]. Patients had to have an IQ of ≥70 based on the clinical judgment of the investigator. The exclusion criteria included clinically significant abnormal laboratory findings, acute or unstable medical conditions, cardiovascular disorder, history of seizures, pervasive developmental disorder, psychosis, bipolar disorder, suicidal ideation, any medical condition that might increase sympathetic nervous system activity, or the need for psychotropic medication other than study drug. Patients already being treated with atomoxetine were also excluded. Other previous treatments were allowed, provided they were discontinued prior to enrolment in the study. The protocol was approved by an ethics committee, and the study was conducted in accordance with the principles of the Declaration of Helsinki. Following a wash-out period, baseline assessments were carried out with all the instruments used. During the first week of treatment, the patients received atomoxetine at a dose of approximately 0.5 mg/kg body weight (BW) per day. During the following 7 weeks, the recommended target dose was 1.2 mg/kg BW per day, but could be adjusted within a range of 0.5–1.4 mg/kg BW per day, depending on effectiveness and tolerability. Medication was given once a day in the morning. Assessments were carried out weekly during the first two weeks of treatment, and every two weeks thereafter. After the 8 week treatment period, the physicians decided in accordance with the patients and their parents whether the patient was to continue treatment for additional 16 weeks. Those who participated in this extension period continued on the same atomoxetine dose which again could be adjusted within a range of 0.5–1.4 mg/kg BW per day as considered appropriate by the physician. During the extension period, three assessments were carried out, after 12, 16, and 24 weeks after baseline. The following instruments were used to assess efficacy: Global Impression of Perceived Difficulties (GIPD), Attention-Deficit/Hyperactivity Disorder Rating Scale (ADHD-RS), Clinical Global Impression-Severity (CGI-S), and the Weekly Rating of Evening and Morning Behavior – Revised (WREMB-R). The results from these scales have been published elsewhere [28,29].

In order to assess the tolerability of atomoxetine in more detail than is possible using spontaneous adverse event reports, the Pediatric Adverse Event Rating Scale (PAERS) [30] was used in the two studies reported here. The data from both studies were combined and analyzed together. Tolerability assessments included monitoring vital signs at every visit and recording all spontaneously reported adverse events, followed by a systematic elicitation of any further adverse events using the PAERS. First, the physician asked an open question as to any adverse events. Then, the patient and the parent (or other primary caregiver) filled out the PAERS independently and without any interference by the physician. Both the patient and the parent had to rate each adverse event in terms of how bothersome or how much of a problem it was during the past week on a scale from 0 (= not present) to 4 (= a lot). If the patient was unable to fill out the scale all by him or her self, an independent person (e. g. a study nurse, but not the parent or physician) was allowed to provide assistance. As the spontaneous adverse event reports captured by the physician preceded the elicitation of any further adverse events using the PAERS, the number of adverse events captured by these two methods potentially differed.

Although the PAERS was designed to measure adverse events, some items are reflecting ADHD symptoms and difficulties associated with ADHD rather than adverse events. These items can be expected to improve but not worsen during ADHD treatment. Of these, all ten items of the PAERS that were thought to reflect emotional well-being by face validity were selected for this post-hoc analysis to measure changes over time.

Noncompliance was defined as missing intake of study drug on more than five consecutive days, failure to take at least 70% of study medication for at least two weeks, or repeated intentional intake of more than the prescribed dose.

### Sample size and statistical analysis

Details on the sample size calculation for the two studies have been published elsewhere [28,29]. The data of all patients were evaluated (Full Analysis Set, FAS) using SAS version 8. The dataset for all analyses of changes from baseline to endpoint consisted of all patients with a baseline measurement and at least one post-baseline measurement during the 8-week treatment phase.

Evaluation was largely descriptive. All tests of statistical significance were carried out at a nominal level of 5% using two-tailed test procedures. Two-sided confidence intervals (CIs) were computed using a 95% confidence level. All inferences regarding statistical significance were based on comparisons of the 95% confidence intervals (CI). This is equivalent to significance tests with p-values and a two-sided α-level of 5%. To avoid correlations of imputed values, only observed cases (OC) analysis were performed. No imputation of missing values like last observation carried forward (LOCF) was applied as the intention was to describe the patterns for patients still on medication.

Spearman's correlation coefficients were computed between all items within each perspective in order to identify patterns of interdependency among items. This analysis was based on all visits. A sensitivity analysis was done using the baseline visit only. 95% confidence intervals for the correlation coefficients were computed based on Fisher's z-transformation.

## Results

### Patient population and disposition

**Figure 1:**
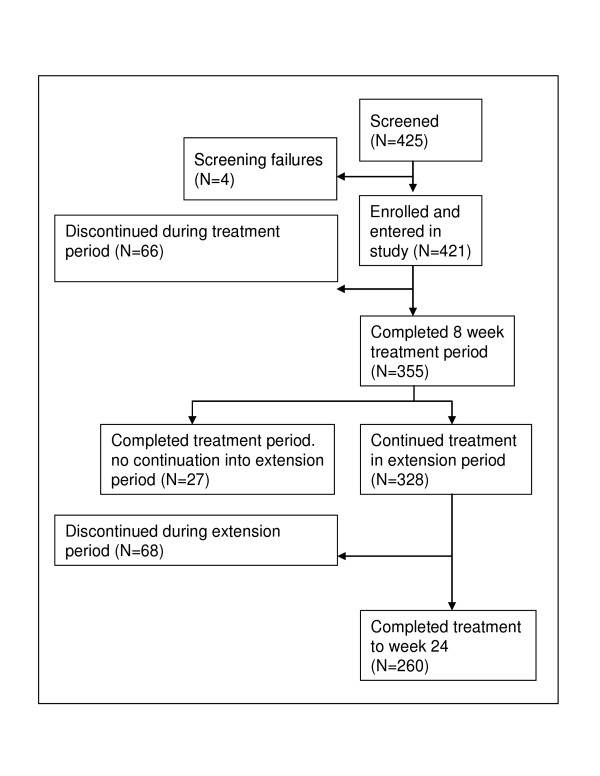
Patient disposition.

Of the 425 patients screened, 421 patients (100%) were enrolled in the two studies and treated with atomoxetine [28,29]. All patients were diagnosed with ADHD according to DSM-IV criteria. The mean age of the patients was 11.1 years, 338 (80.3%) were boys, 83 (19.7%) were girls. The 8-week treatment period was completed by 355 (84.3%) patients. 27 (6.4%) of these did not continue into the extension period because of physician decision. 68 (16.1%) patients discontinued the study between week 8 and week 24. The extension period was completed at week 24 by 260 (61.8%) patients. The reasons for discontinuation at any time during the 24-month observation period were lack of efficacy (12.4%), parent decision (6.9%), adverse event (4.8%), protocol violation 3.6%, patient decision (2.4%), entry criteria exclusion (0.7%), physician decision (0.7%), and patient lost to follow-up (0.5%). The patient disposition is shown in Figure 1.

Table 1 shows the patient characteristics. Boys and patients with the combined subtype ADHD according to DSM-IV [1] tended to be younger and tended to be diagnosed earlier than girls or patients with predominantly inattentive subtype. 239 (70.7%) of the boys and 39 (47.0%) of the girls were diagnosed with the combined subtype. The predominantly inattentive subtype was diagnosed in 86 (25.4%) of the boys and 38 (45.8%) of the girls. The subgroups "predominantly hyperactive-impulsive subtype" and "ADHD, not otherwise specified" were small (6 and 13 individuals, respectively). The mean ADHD-RS total score at baseline was 32.6 [CI 31.5 to 33.6] points. This score decreased to 16.3 [CI 15.1 to 17.5] points at week 8, and was 14.5 [CI 13.1 to 15.8] points at the end of week 24.

**Table 1:** Patient characteristics

		**Age (Years)**		**Age at 1st occurrence of symptoms (Years)**		**Age at 1st ADHD-diagnosis (Years)**	
	**N (%)**	**Mean**	**SD**	**Mean**	**SD**	**Mean**	**SD**
All patients	421 (100)	11.1	2.74	4.0	2.03	8.1	2.59
Boys	338 (80.3)	11.0	2.70	4.0	1.94	7.9	2.54
Girls	83 (19.7)	11.6	2.87	4.3	2.35	8.8	2.70
Combined subtype*	278 (66.0)	10.6	2.58	3.7	1.92	7.6	2.42
Predominantly inattentive subtype*	124 (29.5)	12.4	2.59	4.7	1.93	9.2	2.58
Predominantly hyperactive-impulsive subtype*	6 (1.4)	8.6	2.33	4.2	2.01	6.6	2.22
ADHD, not otherwise specified *	13 (3.1)	11.7	3.08	4.8	3.42	9.6	2.30

* According to the Diagnostic and Statistical Manual of Mental Disorders, Fourth Edition.

Abbreviations: SD = standard deviation.

Pre-existing comorbid conditions were reported for 310 (73.6%) patients, the most frequent being psychiatric comorbidities, specifically conduct disorder (19.7%), oppositional defiant disorder (17.6%), enuresis (4.3%), tic disorder (2.4%), emotional disorder of childhood (2.6%), and depression (1.4%). Physical comorbidities that were reported at a rate of >2% were headache (5.7%), seasonal allergy (4.3%), asthma (3.3%), neurodermatitis (2.6%), acne (2.4%), upper respiratory tract infection (2.1%), and rhinitis (2.1%).

349 (82.9%) of the 421 patients had previously been treated for ADHD. The percentage was similar for the predominantly inattentive subtype (N = 101, 81.5%) and the combined subtype (N = 231, 83.1%). Medications most frequently used before study entry were short-acting methylphenidate (N = 290, 68.9%), long-acting methylphenidate (N = 196, 46.6%), amphetamines (N = 56, 13.3%), antipsychotic drugs (N = 12, 2.9%) and herbal/complementary therapies (N = 10, 2.4%). Commonly reported non-drug therapies prior to study were: occupational therapy (N = 48, 11.4%), "other" psychotherapy (N = 31, 7.4%), structured psychotherapy (N = 42, 10.0%), and remedial education (N = 10, 2.4%). The most frequent reason for discontinuation of previous therapy in patients with pre-treatment was inadequate response (N = 216, 61.9%). N = 68 patients (16.2%) discontinued previous therapy because of adverse events.

The mean atomoxetine dose given during the first week of treatment was 0.50 mg/kg per BW day (SD 0.07, range 0.40 – 0.80 mg/kg BW per day). Thereafter, the mean dose for the respective visit intervals ranged between 1.17 and 1.18 mg/kg per day (min. 0.40, max. 1.50 mg/kg per day). Compliance as defined above was present in 91.2% of all patients over the course of the entire study.

Concomitant medication was taken by 272 (64.6%) of the patients. Cough and cold remedies, analgesics, antibiotics and herbal/complementary medicines were given most frequently. Whilst continuous medication with any psychotropic compound other than the study medication led to discontinuation of the patient in the study, 3.8% (N = 16) of patients did receive a psychotropic medication at least once over the entire course of the 24-week study. The medication included compounds such as St. John's Wort, imipramine or a benzodiazepine. Concomitant behavioral therapy was given to 27 (6.4%) patients, and 20 (4.8%) patients received additional occupational therapy.

### Results from ten items of the PAERS

The following ten items of the Pediatric Adverse Event Rating Scale (PAERS) were selected to investigate emotional well-being: "feeling withdrawn or numb" (item 8), "nervous, tense, or uptight" (item 16), "trying to hurt him or her self" (item 20), "feeling restless or keyed up" (item 26), "sad or low mood/unhappy" (item 32), "drowsy or 'out of it"' (item 37), "unusually good mood/super happy" (item 38), "not interested/no enthusiasm" (item 39), "angry or irritable/in a bad mood" (item 42), and "thinking about or wanting to hurt self" (item 43). Each of these items was rated from three perspectives (patient, parent, and physician) like all PAERS items. If present, the severity of the respective behavior or emotional state was rated on a 5 point Likert scale (0 = not present, 1 = mild, 2 = moderate, 3 = severe, and 4 = extreme).

The following five groups of items were identified, whose correlations were larger than 0.4 between items: (a) items relating to depressed mood (items 8, 32, and 39), (b) items relating to self-harm (items 20 and 43), (c) items relating to irritability/agitation (items 16, 26, and 42), (d) one item relating to drowsiness (item 37), and (e) one item relating to euphoria (item 38) (Table 2). The correlation of the various items in the five groups is shown in Table 3.

**Table 2:** Groups of items of the Pediatric Adverse Event Rating Scale (PAERS) used to assess emotional well-being

**Item No.**	**Item Groups**
**Items relating to depressed mood**	
8	Feeling withdrawn or numb
32	Sad or low mood/unhappy
39	Not interested/no enthusiasm
**Items relating to self-harm**	
20	Trying to hurt him or her self
43	Thinking about or wanting to hurt self
**Items relating to irritability/aggression**	
16	Nervous, tense, or uptight
26	Feeling restless or keyed up
42	Angry or irritable/in a bad mood
**Item relating to drowsiness**	
37	Drowsy or "out of it"
**Item relating to euphoria**	
38	Unusually good mood/super happy

**Table 3:** The five groups of items from the Pediatric Adverse Event Rating Scale (PAERS) whose Spearman's correlation coefficients for the physician rating were larger than 0.4 between items (all visits pooled; 3611 observations).

**Items relating to depressed mood (items 8, 32, and 39)**
*a. Correlation of items, physician rated*
item 8 vs. item 32 r = 0.448 (95% CI 0.421 to 0.473)
item 8 vs. item 39 r = 0.413 (95% CI 0.385 to 0.439)
item 32 vs. item 39 r = 0.465 (95% CI 0.439 to 0.490)
*b. Correlation of items, parent rated*
item 8 vs. item 32 r = 0.534 (95% CI 0.510 to 0.557)
item 8 vs. item 39 r = 0.424 (95% CI 0.396 to 0.450)
item 32 vs. item 39 r = 0.444 (95% CI 0.417 to 0.470)
*c. Correlation of items, patient rated*
item 8 vs. item 32 r = 0.339 (95% CI 0.309 to 0.367)
item 8 vs. item 39 r = 0.313 (95% CI 0.283 to 0.342)
item 32 vs. item 39 r = 0.383 (95% CI 0.355 to 0.411)
**Items relating to self-harm (items 20 and 43)**
*d. correlation of items, physician rated*
item 20 vs. item 43 r = 0.606 (95% CI 0.585 to 0.626)
*e. correlation of items, parent rated*
item 20 vs. item 43 r = 0.659 (95% CI 0.640 to 0.677)
*f. correlation of items, patient rated*
item 20 vs. item 43 r r = 0.598 (95% CI 0.576 to 0.618)
**Items relating to irritability/agitation (items 16, 26, and 42)**
*g. Correlation of items, physician rated*
item 16 vs. item 26 r = 0.478 (95% CI 0.453 to 0.503)
item 16 vs. item 42 r = 0.459 (95% CI 0.433 to 0.484)
item 26 vs. item 42 r = 0.471 (95% CI 0.445 to 0.496)
*h. Correlation of items, parent rated*
item 16 vs. item 26 r = 0.539 (95% CI 0.515 to 0.562)
item 16 vs. item 42 r = 0.516 (95% CI 0.492 to 0.540)
item 26 vs. item 42 r = 0.533 (95% CI 0.509 to 0.556)
*i. Correlation of items, patient rated*
item 16 vs. item 26 r = 0.367 (95% CI 0.338 to 0.395)
item 16 vs. item 42 r = 0.353 (95% CI 0.324 to 0.381)
item 26 vs. item 42 r = 0.379 (95% CI 0.350 to 0.406)
**Item relating to drowsiness (item 37)**
**Item relating to euphoria (item 38)**

In general, the correlations were moderate to high only for parent and physician ratings, but not for patient ratings. The pattern of moderate to high correlations was similar between parent and physician ratings. In the sensitivity analyses using baseline ratings only (rather than all ratings), the correlations were generally lower, but confirmed the overall pattern of correlations based on the other points in time. A total score of all the items was not calculated as correlations were low between items that belonged to different groups (Table 2). These correlations are not reported here.

### Items relating to depressed mood

Based on the confidence intervals at baseline, the parent ratings of the items "feeling withdrawn or numb" (item 8), "sad or low mood/unhappy" (item 32), and "not interested/no enthusiasm" (item 39), were significantly higher compared to both the patients and the physician ratings, which were similar. However, mean scores for all items were below 0.81 (Table 4). The scores for the items relating to depressed mood decreased over time. The mean change from baseline was statistically significant for all three items and all three perspectives both at week 8 and at week 24 (Table 4). Generally, there was a tendency for mean scores to decrease further the longer patients stayed on medication. Moreover, the decrease in scores was generally most pronounced in parent ratings, followed by patient and physician ratings.

**Table 4:** Baseline ratings and change from baseline at weeks 8 and 24

**Item**	**Week**	**Physician rating**			**Parent rating**			**Patient rating**		
		**Mean**	**95% CI**	**SD**	**Mean**	**95% CI**	**SD**	**Mean**	**95% CI**	**SD**
***Items relating to depressed mood***										
**8**	0	0.32	0.26 to 0.39	0.71	0.54	0.45 to 0.63	0.93	0.33	0.26 to 0.40	0.75
	Ch 8	-0.14	-0.22 to -0.05	0.77	-0.25	-0.35 to -0.15	0.96	-0.20	-0.28 to -0.12	0.76
	Ch 24	-0.20	-0.29 to -0.11	0.71	-0.33	-0.45 to -0.21	0.97	-0.23	-0.32 to -0.13	0.75
**32**	0	0.50	0.42 to 0.58	0.83	0.81	0.71 to 0.91	1.04	0.44	0.34 to 0.53	0.95
	Ch 8	-0.18	-0.28 to -0.09	0.87	-0.31	-0.43 to -0.19	1.08	-0.23	-0.34 to -0.12	0.99
	Ch 24	-0.26	-0.37 to -0.15	0.90	-0.44	-0.57 to -0.31	1.10	-0.15	-0.28 to -0.02	1.03
**39**	0	0.48	0.40 to 0.57	0.91	0.78	0.68 to 0.89	1.07	0.47	0.38 to 0.56	0.94
	Ch 8	-0.17	-0.27 to -0.07	0.90	-0.28	-0.40 to -0.16	1.11	-0.28	-0.38 to -0.18	092
	Ch 24	-0.28	-0.39 to -0.17	0.90	-0.36	-0.49 to -0.22	1.14	-0.31	-0.43 to -0.19	1.01
***Items relating to self-harm***										
**20**	0	0.07	0.03 to 0.10	0.38	0.11	0.06 to 0.16	0.51	0.08	0.04 to 0.11	0.40
	Ch 8	-0.01	-0.05 to 0.03	0.37	-0.02	-0.07 to 0.02	0.43	0.00	-0.05 to 0.06	0.51
	Ch 24	-0.02	-0.06 to 0.02	0.32	-0.03	-0.09 to 0.02	0.42	-0.02	-0.06 to 0.02	0.34
**43**	0	0.07	0.03 to 0.10	0.37	0.11	0.06 to 0.16	0.52	0.08	0.04 to 0.12	0.40
	Ch 8	-0.02	-0.07 to 0.03	0.44	-0.01	-0.06 to 0.04	0.47	0.02	-0.02 to 0.06	0.37
	Ch 24	-0.02	-0.06 to 0.03	0.37	-0.03	-0.08 to 0.01	0.39	0.00	-0.05 to 0.05	0.42
***Items relating to irritability/agitation***										
**16**	0	0.98	0.87 to 1.08	1.10	1.27	1.16 to 1.39	1.20	0.61	0.51 to 0.70	1.01
	Ch 8	-0.68	-0.80 to -0.55	1.17	-0.78	-0.91 to -0.65	1.18	-0.36	-0.48 to -0.25	1.07
	Ch 24	-0.69	-0.83 to -0.55	1.16	-0.80	-0.94 to -0.65	1.19	-0.41	-0.54 to -0.27	1.10
**26**	0	1.41	1.30 to 1.53	1.22	1.79	1.67 to 1.92	1.27	0.66	0.56 to 0.77	1.09
	Ch 8	-0.97	-1.10 to -0.83	1.24	-1.09	-1.24 to -0.95	1.34	-0.42	-0.52 to -0.31	1.00
	Ch 24	-0.93	-1.07 to -0.79	1.18	-1.10	-1.26 to -0.94	1.31	-0.43	-0.56 to -0.30	1.08
**42**	0	1.32	1.20 to 1.45	1.26	2.00	1.88 to 2.12	1.22	0.93	0.81 to 1.04	1.22
	Ch 8	-0.52	-0.64 to -0.39	1.17	-0.72	-0.86 to -0.57	1.34	-0.43	-0.57 to -0.29	1.26
	Ch 24	-0.55	-0.70 to -0.41	1.18	-0.85	-1.02 to -0.68	1.38	-0.46	-0.62 to -0.30	1.33
***Item relating to drowsiness***										
**37**	0	0.25	0.19 to 0.30	0.61	0.43	0.35 to 0.50	0.81	0.40	0.32 – 0.48	0.85
	Ch 8	-0.08	-0.15 to 0.00	0.71	-0.16	-0.25 to -0.07	0.83	-0.16	-0.26 to -0.07	0.88
	Ch 24	-0.17	-0.24 to -0.10	0.59	-0.25	-0.36 to -0.14	0.88	-0.20	-0.31 to -0.09	0.86
***Item relating to euphoria***										
**38**	0	0.35	0.28 to 0.43	0.77	0.65	0.55 to 0.74	1.00	1.06	0.93 to 1.19	1.33
	Ch 8	-0.14	-0.22 to -0.05	0.83	-0.32	-0.43 to -0.20	1.05	-0.40	-0.56 to -0.25	1.41
	Ch 24	-0.15	-0.25 to -0.06	0.75	-0.40	-0.51 to -0.29	0.92	-0.43	-0.58 to -0.28	1.24

Items: 8 = feeling withdrawn or numb; 32 = sad or low mood/unhappy; 39 = not interested/no enthusiasm; 20 = trying to hurt him or her self; 43 = thinking about or wanting to hurt self; 16 = nervous, tense, or uptight, 26 = feeling restless or keyed up; 42 = angry or irritable/in a bad mood; 37 = drowsy or "out of it, 38 = unusually good mood/super happy. Abbreviations: 0 = Week 0 (Baseline); Ch 8 = change to week 8, Ch 24 = change to Week 24; CI = confidence interval; SD = standard deviation.

### Items relating to self-harm

The scores for the items relating to self-harm were extremely low at baseline and stayed low over time (Table 4). The scores were comparable in terms of the three perspectives. No significant changes were observed compared to baseline.

### Items relating to irritability/agitation

At baseline, a similar pattern was observed for items "nervous, tense, or uptight" (item 16), "feeling restless or keyed up" (item 26), and "angry or irritable/in a bad mood" (item 42). Based on the confidence intervals, the parent rating was significantly higher than the physician rating, which was again significantly higher than the patient rating for all three items. The scores for the items relating to irritability/agitation decreased over time (Table 4). The decreases from baseline were largest for parents, followed by physicians and patients: the mean changes from baseline were statistically significant for all perspectives, all three items, and both at week 8 and week 24, as shown by non-overlapping confidence intervals.

### Item relating to drowsiness

Based on the confidence intervals at baseline, the item "drowsy or 'out of it"' (item 37) was scored similarly by parents and patients, and significantly higher than by physicians. The scores for the item relating to drowsiness decreased over time (Table 4). Also the changes from baseline were more pronounced in parent and patient ratings than in physician ratings. The mean changes from baseline were statistically significant for all perspectives and both at week 8 and week 24, as shown by non-overlapping confidence intervals.

### Item relating to euphoria

Based on the confidence intervals at baseline, the item "unusually good mood/super happy" (item 38) was scored significantly higher by patients than by parents, and significantly higher than by physicians. The scores for the item relating to euphoria decreased over time (Table 4). The changes from baseline were scored similarly by patients and physicians, but the decreases were smaller in the physician rating. The mean changes from baseline were statistically significant in terms of all three perspectives and were significant both at week 8 and week 24, as shown by non-overlapping confidence intervals.

## Discussion

The aim of this post-hoc analysis was to evaluate the scores of the ten items of the Pediatric Adverse Event Rating Scale (PAERS) that were considered to be related to emotional well-being by face validity. Each of these ten items was rated from three perspectives: the patient, the parent, and the physician perspective. These perspectives were subsequently compared in terms of the height of scores and changes in the scores over time.

Emotional well-being as reflected by the scores on the respective ten items of the PAERS showed both similarities as well as differences both regarding the course of the scores over time and comparisons between the three perspectives (patient, parent, physician). Generally, scores for all items rated from all three perspectives decreased over the 24-week duration of the two studies. Thus, emotional well-being as reflected by the scores on the ten items of the PAERS was seen to improve during treatment with atomoxetine.

Correlations between parent and physician scores were generally higher than correlations between parent and patient scores as well as correlations between physician and patient scores. There were, however, distinct differences between the patterns observed for the five groups of items relating to depressed mood, self-harm, irritability/agitation, drowsiness, and euphoria.

For the items relating to depressed mood, parent ratings resulted in higher scores than either patient or physician ratings at baseline. This may be due to parents being particularly concerned about the emotional well-being of their children. Over time, however, there is a reduction in the scores for these items from all three rater perspectives. Obviously, all individuals concerned recognize an improvement in the emotional well-being of the patients over time. Surprisingly, both patient and physician ratings on the PAERS were similar, although children and adolescents seemed to dissimulate their difficulties or failed to perceive their difficulties correctly according to the Global Impression of Perceived Difficulties (GIPD), whilst physicians perceived the child's difficulties as being considerably greater [28,29]. Mean changes for most items and ratings were approximately one third of a standard deviation (Table 4). This can be considered a moderate change, given the low scores at baseline.

For the items relating to self-harm, scores from all three perspectives were very low at baseline and did not change significantly whilst the child or adolescent is being treated with atomoxetine. Mean changes from baseline for these items and ratings were negligible. This finding is encouraging, because it suggests that attempts to self-harm or thoughts of self-harm are not aggravated by treatment with atomoxetine.

For the items relating to irritability/agitation, scores differed significantly depending on the rater. Whilst baseline scores rated by parents were the highest, baseline scores rated by patients were lowest, and baseline scores rated by physicians were in between. These findings may be due to the high impact that a child's irritability/agitation may have on the parents. The physician may be less likely to observe irritability/agitation than a parent might, and children or adolescents seemed to dissimulate their difficulties in these two studies [28,29]. Mean changes for most items in this dimension and for physician and parent ratings were greater than one half of the standard deviation (Table 4). This is a considerable change. In contrast, the patient ratings for these items changed to a smaller degree. This finding may be a result of the lower patient-rated scores at baseline.

The scores for the item relating to drowsiness as rated by patients and parents were similar whilst the scores rated by the physicians differed from the scores rated either by the patients or the parents at baseline. Physician-rated baseline scores were lower, which may be due to the physicians not having as much opportunity to witness any drowsiness as parents may do. Patients can be expected to experience this well-known adverse event related to atomoxetine [36]. Mean changes for this item were just below one third of the standard deviation. This can be considered a moderate change, given the low scores at baseline.

The scores for the item relating to euphoria differed between all three perspectives (patient, parent, physician). Whilst patient ratings resulted in the highest scores for euphoria, the scores from physician ratings were the lowest and scores from parent ratings were in between. The greater euphoria experienced by the patients compared to the euphoria seen by the parents or physicians seems to correspond to the lower degree of ADHD-related difficulties perceived by patients compared to the parent or physician perspectives as measured by the GIPD in these two studies [28,29]. The rating of euphoria by the parents may have resulted in scores that more objectively reflect the actual situation, whilst the physicians may not have had adequate opportunity to witness the euphoria before carrying out their rating. Mean changes for this item were approximately one third of the standard deviation. This can be considered a moderate change, given the low to moderate scores at baseline.

This study has several limitations. Most importantly, the study did not include a placebo control, so that the degree to which the results reflect drug-specific effects cannot be determined definitely. More specifically, placebo-controlled studies would be needed to distinguish direct medication effects on emotional well-being from indirect effects caused by improvement of core symptoms. Furthermore, the age-distribution of the sample does not reflect the age-distribution of individuals with ADHD in the general pediatric population. This is due to the fact that this analysis is based on two studies, one in children and one in adolescents. Whilst the age-distribution is normal within each of the two otherwise identical studies, the age-distribution of the combined samples is not quite normal, as it shows two peaks. Due to the open-label design, unspecific factors such as rater bias, expectation effects, and time effects cannot be ruled out. However, this does not automatically compromise the validity of the results [37]. Furthermore, although both mean symptom reduction and improvement in emotional well-being were considerable, the results do not allow direct comparison against changes of these parameters upon treatment with other ADHD medications. Treatment emergent adverse events occurring in the two studies on which this analysis is based have been reported and discussed elsewhere in more detail [28,29]. For evaluating the adverse event profile, it needs to be taken into account that only those patients for whom the physician decided to continue atomoxetine treatment at week 8 were followed for additional 16 weeks until week 24.

Taken together, these findings could be expected, as cognition and the regulation of emotion are known to influence one another [38]. Furthermore, cognitive control of emotion involves frontal structures of the brain [39], areas of the brain that play an important role in the pathophysiology of ADHD [3]. Thus, any pharmacological treatment that is effective on the core symptoms of ADHD and executive functioning can also be expected to improve the emotional regulation and thus the emotional well-being of patients with ADHD. This hypothesis is supported by the findings from this secondary analysis. These findings appear particularly relevant in face of the important role that emotional regulation plays in children, adolescents, and adults with ADHD [4,5,39-43].

## Competing interests

Research was funded by Lilly Deutschland GmbH, Bad Homburg, Germany. Peter M. Wehmeier, Ralf W. Dittmann and Alexander Schacht are full-time employees of Lilly Deutschland GmbH.

## Authors' contributions

PMW, RWD, ML and AS developed the two clinical trials, SGS and JSM developed the PAERS scale, ML had the idea and AS developed the analyses for this manuscript. All authors participated in the interpretation of data, PMW and AS drafted the manuscript, RWD, SGS, ML and JSM revised it critically for important intellectual content. All authors read and approved the final manuscript.
